# Deciphering the importance of culture pH on CD22 CAR T-cells characteristics

**DOI:** 10.1186/s12967-024-05197-5

**Published:** 2024-04-24

**Authors:** Michaela Prochazkova, Alexandra Dreyzin, Lipei Shao, Pam Garces, Yihua Cai, Rongye Shi, Alejandra Pelayo, Yong Soo Kim, Victoria Pham, Sue Ellen Frodigh, Shannon Fenton, Catherine Karangwa, Yan Su, Kathryn Martin, Nan Zhang, Steven L. Highfill, Robert P. Somerville, Nirali N. Shah, David F. Stroncek, Ping Jin

**Affiliations:** 1 Center for Cellular Engineering, Department of Transfusion Medicine, Clinical Center, National Institutes of Health, Bethesda, MD USA; 2 Center for Cancer and Blood Disorders, Children’s National Hospital, Washington, DC USA; 3 Pediatric Oncology Branch, Center for Cancer Research, National Cancer Institute, National Institutes of Health, Bethesda, MD USA

**Keywords:** Chimeric antigen receptor (CAR) T-cells, pH, Clinical manufacturing, Immunotherapy

## Abstract

**Background:**

Chimeric antigen receptor (CAR) T-cells have demonstrated significant efficacy in targeting hematological malignancies, and their use continues to expand. Despite substantial efforts spent on the optimization of protocols for CAR T-cell manufacturing, critical parameters of cell culture such as pH or oxygenation are rarely actively monitored during cGMP CAR T-cell generation. A comprehensive understanding of the role that these factors play in manufacturing may help in optimizing patient-specific CAR T-cell therapy with maximum benefits and minimal toxicity.

**Methods:**

This retrospective study examined cell culture supernatants from the manufacture of CAR T-cells for 20 patients with B-cell malignancies enrolled in a phase 1/2 clinical trial of anti-CD22 CAR T-cells. MetaFLEX was used to measure supernatant pH, oxygenation, and metabolites, and a Bio-Plex assay was used to assess protein levels. Correlations were assessed between the pH of cell culture media throughout manufacturing and cell proliferation as well as clinical outcomes. Next-generation sequencing was conducted to examine gene expression profiles of the final CAR T-cell products.

**Results:**

A pH level at the lower range of normal at the beginning of the manufacturing process significantly correlated with measures of T-cell expansion and metabolism. Stable or rising pH during the manufacturing process was associated with clinical response, whereas a drop in pH was associated with non-response.

**Conclusions:**

pH has potential to serve as an informative factor in predicting CAR T-cell quality and clinical outcomes. Thus, its active monitoring during manufacturing may ensure a more effective CAR T-cell product.

**Supplementary Information:**

The online version contains supplementary material available at 10.1186/s12967-024-05197-5.

## Background

Chimeric antigen receptor (CAR) T-cell therapy has been highly effective in patients with B-cell hematologic malignancies, including acute lymphoblastic leukemia [1–5] and non-Hodgins’ lymphoma [6–8], and ongoing research aims to expand applications to solid tumors [9, 10] and autoimmune diseases [11–13]. Since the initial approval of the CD19-targeted CAR T-cell, tisagenlecleucel (Kymriah), in 2017, the US Food and Drug Administration (FDA) has approved five more CAR T-cell therapies for acute lymphoblastic leukemia, non-Hodgkin lymphoma, and multiple myeloma. The rapid growth of this therapy is illustrated by the more than 700 CART trials actively recruiting patients (clinicaltrials.gov, February 2024).

As new CAR T-cell therapies become commercialized and enter into clinical use, it is essential to produce CAR T-cells that are consistently of high quality and potency. With more clinical centers aiming to establish point-of-care processes of CAR T-cell manufacturing, maintaining consistent cell culture conditions will become increasingly challenging.

Cell culture is a dynamic process, so understanding the interactions between cells, culture media, nutrients, and growth factors is critical for producing high quality and potent cell therapy products. Cell counts, viability, and expansion are routinely monitored during CAR T-cell manufacturing, but little attention is paid to variables such as metabolites, oxygen and carbon dioxide tension, or pH. These factors, however, may be associated with CAR T-cell proliferation and fitness. The pH in particular is a simple indicator that may reflect carbon dioxide tension, lactate accumulation, and rate of T-cell proliferation. Thus, monitoring these variables could be valuable in predicting properties of the final cell therapy product.

Not only could culture media characteristics serve as indicators of T-cell expansion, but they could also be a potential point of intervention. The pH of the culture media has been shown to affect T-cell proliferation, differentiation, and metabolism. T-cells cultured in acidic media (pH 6.6 and lower) have reduced activation, proliferation, and slower differentiation to antigen-specific cytotoxic T-lymphocytes [14, 15]. Moreover, in a study of tumor-infiltrating T-lymphocytes, acidic media led to suppression of T-cell function, including reduced cytokine secretion and lower expression of T-cell receptors [16]. On the other hand, while prolonged (12 days) ex vivo expansion of T-cells in an acidic environment (pH 6.6) reduced rate of proliferation, it also promoted CD8+ T-cell stemness [17]. Additionally, this extended expansion of T-cells in acidic media triggered T-cell metabolic reprogramming with restricted glycolysis and increased long chain fatty acid metabolism [17]. Studies of how changes in culture media affect T-cells in the context of CAR T-cell production specifically are limited.

Accurate monitoring and control of pH and metabolites may be an important opportunity to ensure optimal CAR T-cell performance and function. However, these factors are rarely monitored during clinical Good Manufacturing Practice (GMP) CAR T-cell generation and their effect on CAR T-cell growth and differentiation has yet to be fully investigated. In this study, we examined the pH and metabolic parameters of anti-CD22 CAR T-cell cultures and explored the relationship between culture conditions and clinical outcomes.

## Methods

### Patients’ cohort

The products included in this study were derived from a cohort of 20 children and young adults who were diagnosed with recurrent or refractory CD22-expressing B-cell malignancies and were enrolled on a Phase 1/2 clinical trial of anti-CD22 CAR T-cells (NCT 02315612). All patients were treated in the Pediatric Oncology Branch of the National Cancer Institute, National Institutes of Health at a dose level of 0.3 × 10^6^ cells.

### CD22 CAR-T manufacturing process

All anti-CD22 CAR T-cell products were manufactured at the Center for Cellular Engineering (CCE) between September 2019 and August 2022. The CCE is compliant with good manufacturing practice (GMP) and good clinical laboratory practice (GCLP) standards and has manufactured numerous CAR T-cell products for phase 1 and 2 clinical trials at the National Institutes of Health. All the products were manufactured using autologous mononuclear cells collected by apheresis. T-cells from fresh (n = 5) or cryopreserved (n = 15) autologous mononuclear cells were selected on CliniMACS (Miltenyi Biotec) using anti-CD4 and anti-CD8 magnetic beads and stimulated with anti-CD3/CD28 Dynabeads (ThermoFisher) with a ratio of 1 cell:3 beads. Cells were seeded into culture bags at a density of 1.5–2.0 × 10^6^ viable CD3^+^ cells. Cell culture was maintained in AIM-V medium (Gibco) containing heat inactivated human AB serum (Valley Biomedical), Glutamax (Gibco) and IL2 (Aldesleukin) in a 37 °C, 5% CO_2_ incubator for 9 days. On day 2 of the culturing process, cells were transduced with GMP grade CD22 lentiviral vector (Lentigen) using bag spinoculation (1000 g for a period of 2 h). On day 3 (D3), transduction was stopped by media exchange. Debeading and cell density adjustment to 0.4 × 10^6^ viable cells/mL was performed on day 4 (D4) of the culturing process. Cell density was further adjusted on Day 7 (D7) to 0.6–1 × 10^6^ viable cells/mL. All cultures were harvested on Day 9 (D9) and were either freshly infused (n = 7) or cryopreserved (n = 9). A simplified 9-day manufacturing process is illustrated in Additional file 1: Supplementary Figure 1a.

### Collection of culture media supernatants

Culture media supernatants were collected at four different time points during the CD22 CAR T-cell culture (on Day 2, 3, 4 and 9). 10 mL of culture supernatant was collected before transduction on D2, when transduction was stopped on D3, and before debeading on D4. Final supernatants were collected during the cell harvest on D9. All supernatants were aliquoted (1 mL per vial), immediately frozen and stored at − 80 °C until further analysis.

### Metabolite analysis

A Vi-CELL MetaFLEX bioanalyzer (Beckman Coulter) was used to measure pH, glucose, lactate, pCO_2_ and pO_2_ levels during the manufacturing process. At the time of the analysis, supernatants collected at various time-points during the culturing process were thawed and warmed to 24 °C. 65 uL of temperature adjusted supernatants were loaded into the MetaFLEX device for testing.

### Bio-Plex assay

Culture media cytokine, chemokine, and growth factor levels were measured at 2 different time points (D2 and D9) during the culture process using the Bio-Plex 200 system (Bio-Rad Laboratories). Bio-Plex assays were performed according to the manufacturers’ instructions using commercially available Bio-Plex Pro Human Cytokine Screening 48-plex Panel Assay. All assay values are reported in pg/mL.

### Bulk mRNA sequencing

We have previously described the bulk RNA sequencing procedures in detail [18]. In brief, total mRNA from CAR T-cell products was isolated using the miRNeasy Mini Kit. The concentration and quality were assessed using Nanodrop 8000 and 2100 Bioanalyzer, respectively. DNA libraries were prepared using the TruSeq Stranded Total RNA kit and sequenced on the Illumina Nextseq550 platform.

### Transcriptome data analysis

Raw fastq files underwent quality control using FastQC and were further processed with Trimmomatic to remove adapter sequences and low-quality reads. After filtering, the reads were aligned to the human reference genome (GENCODE hg38) using the STAR aligner. Gene expression levels were quantified using subread (featureCounts), and TPM values were utilized for downstream analysis. For differential expression analysis, we employed the limma package in RStudio with custom scripts. Threshold for definition of differentially expressed genes (DEGs) is p value < 0.01 & |FoldChange|≥ 2. We then performed gene set enrichment analysis (GSEA), a bioinformatic method to determine whether a predefined set of genes showed statistically significant, concordant differences between two biological states. We divided the gene expression from our RNA-seq data into two sets: up-regulated and down-regulated set. Subsequently, we examined the presence of genes associated with each pathway in either the up-regulated or down-regulated set. The degree of enrichment was quantified by a normalized enrichment score (NES). A significant positive NES value suggests that members of the selected pathway gene set tend to appear in the up-regulated set, whereas a significant negative NES value indicates the opposite scenario. The analysis was conducted by the built-in function of the cluster Profiler package [19] in RStudio.

### Flow cytometry

Transduction efficiency, viability and percentage of cells expressing CD3, CD4, CD8, CD14, CD15, and CD56 were measured using flow cytometry following staining with fluorescently labelled antibodies (BD Biosciences). The CD22-CAR vector transduction efficiency was detected using protein L (ThermoFisher). Untransduced cells were used to determine the positive gate for transduction efficiency. The gating strategy of protein L expression on CD22 CAR T-cells is displayed in Additional file 1: Supplementary Figure 1c. Frequencies of the above-mentioned markers were acquired on the BD FACS CantoII (BD Biosciences). Thirty thousand events in total were collected for each sample. Data were analyzed using BD FACSDiva software.

### Statistical analysis

All data are presented as mean ± SEM. Graphical and statistical analysis was performed using GraphPad Prism 7 software. Statistical differences between the groups were assessed either using an unpaired *t* test, Mann–Whitney, or nonparametric Spearman correlation. P value less than 0.05 was considered significant, except for the next-generation sequencing data analysis.

## Results

### Overview of CD22 CAR-T cell expansion during the manufacturing process

The clinical characteristics of patients whose CD22 CAR T-cell products were included in the study are detailed in Table 1. In total, twenty CD22 CAR T products were analyzed. All 20 products met the 0.3 × 10^6^/kg target dose. Four products were not infused, as the patients experienced rapidly progressive disease during the manufacturing process. Seven patients received infusions of fresh CAR T-cells, and nine patients received cryopreserved CAR T-cells.

**Table 1:** Patients and manufacturing process characteristics

Number of patients	20	
Disease	B cell malignancies (B-ALL in 19 patients, DLBCL in 1 patient)	
Age (mean, years)	13	
Weight (mean, kg)	45.9	
Sex	8F/12M	
Race	White	13
	Asian	2
	AA	2
	UNK	2
	Multiple	1
Ethnicity	NH	14
	H	6
Prior HSCT	10/20	
Prior CAR T-cell therapy	15/20	
Expansion method	Bag culture	
Dose	0.3 × 10^6^/kg	
Starting material	CliniMACS enriched CD4/CD8	
Media and cytokines	AimV, IL2	
Stimulation	CD3/CD28 Dynabeads	
Length of the culture	9 days	
Vector	Lentivirus	

*B-ALL* B-cell acute lymphoblastic leukemia, *DLBCL* diffuse large B cell lymphoma, *F* Female, *M* Male, *AA* Afro-American, *UNK* Unknown, *NH* non-Hispanic, *H* Hispanic, *HSCT* hematopoietic stem cell transplant

**Fig. 1:**
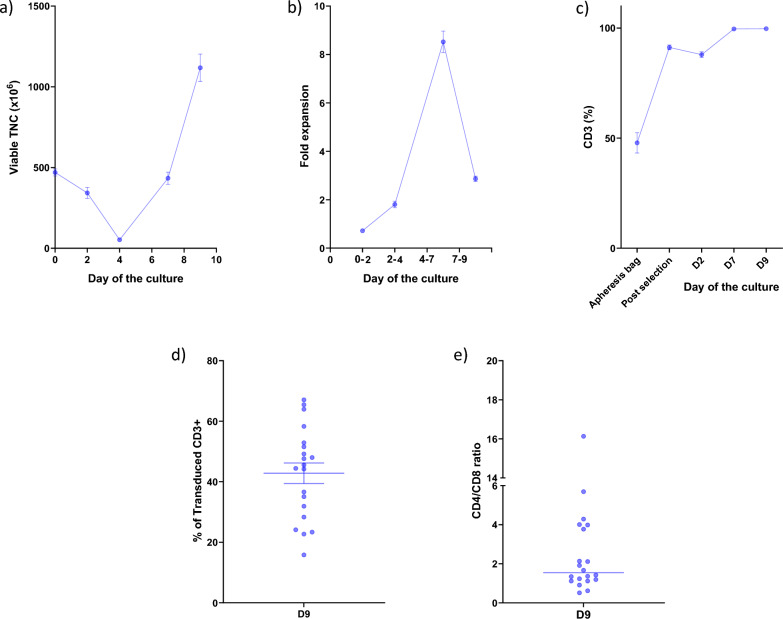
Characteristics of twenty CD22 CAR T-cell products manufactured at CCE of the NIH Clinical Center. **a** Viable total nucleated cells (TNC) count at the start of the CD22 manufacturing process (470 × 10^6^) doubled over the time of the 9-day culturing process to 1.1 × 10^9^ of viable TNC at the harvest. **b** Fold expansion rate reached its maximum on D7 of the manufacturing process (8.5) with a drop at the harvest (2.9). **c** CD3 percentage increased over the culturing process from 48% at apheresis bag to 99.7% of CD3^+^ at the harvest. **d** Transduction efficiency measured via flow cytometry reached 43% of protein L^+^ cells at the culture harvest. **e** CD4/CD8 ratio at the harvest

During the manufacturing process, viable total nucleated cells (TNC) were counted on days 0, 2, 4, 7 and 9. The mean TNC at the start of the manufacturing process was 470 × 10^6^ cells (range 288–565 × 10^6^). On Day 2, a mean of 343 × 10^6^ total nucleated cells underwent transduction with lentiviral vector. On Day 4, there was a drop in total nucleated cells with a mean of 52 × 10^6^ cells (range 26–76 × 10^6^). The total number of cells harvested on Day 9 was 1.1 × 10^9^ (range 0.3–1.7 × 10^9^, Fig. 1a). Fold expansion was determined between the following days of the cell culture: 0–2, 2–4, 4–7 and 7–9. The maximum fold expansion was achieved on Days 4–7 of the culturing process (mean 8.5, range 4.6–11.9) with a decrease in expansion between Days 7 and 9 (mean 2.9, range 2.0–3.9, Fig. 1b). As shown in Fig. 1c, the percentage of CD3^+^ cells increased from 47.9 in the apheresis bag to 91.2 post-CD4/CD8 selection to 99.7 at the time of the harvest of CAR T-cells. The apheresis bag and selected CD4/CD8 T-cells were analyzed for additional markers such as CD56, CD14, or CD15. Additional file 1: Supplementary Figure 1b shows the analysis of these markers. The transduction efficiency (percent of protein L^+^ cells) was assessed on Days 7 and Day 9 of the manufacturing process. On Day 7, after the fastest period of expansion, 31% transduction was observed (data not shown). By Day 9, a significantly larger proportion of cells expressed protein L, (42.8%, p = 0.0073, Fig. 1d), suggesting preferential expansion of transduced cells. The final CD4/CD8 ratio was 2.8 (Fig. 1e).

### The pH of the cell culture at the beginning of the manufacturing process correlated with CAR T-cell growth, expansion, and metabolic activity

**Fig. 2:**
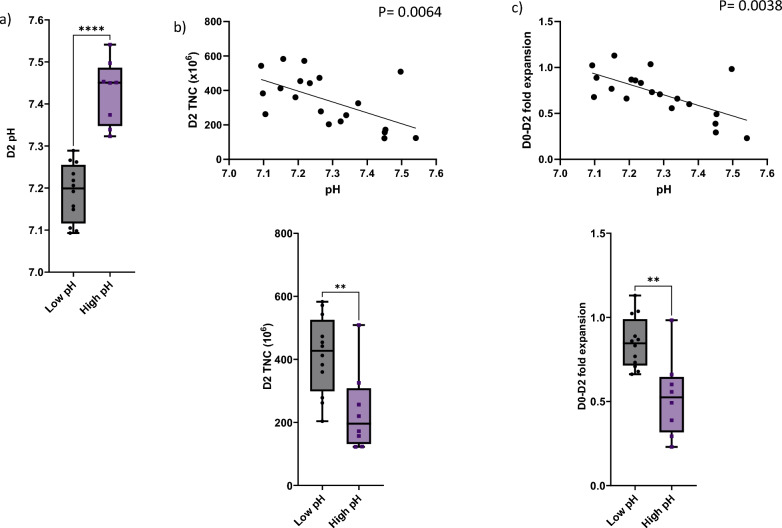

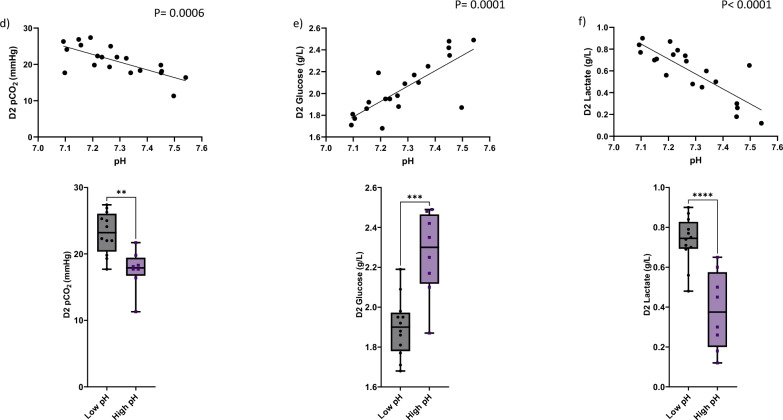
Extracellular changes in pH induced TNC, fold expansion and metabolites change at the beginning of CD22 CAR T-cells manufacturing process. **a** 12 samples were preserved in low pH (mean 7.2) whereas 8 samples in high pH (mean 7.4, p < 0.0001). **b** Higher pH negatively correlated and reduced number of total nucleated cells (236 × 10^6^) on Day 2 of the manufacturing process in comparison to 414 × 10^6^ cultured in lower pH (p < 0.0061). **c** pH values significantly correlated with T-cell expansion. T cells cultured in lower pH reached significantly higher expansion rate related to the ones sustained in high pH (0.85 vs 0.53, p < 0.0015). **d** Tension of carbon dioxide significantly correlated with pH and was decreased in high pH (23.2 vs 17.6 mmHg, p < 0.001). **e** Low pH induced drop in glucose levels (2.3 vs 1.9 g/L, p < 0.003) whereas opposite trend was seen for lactate levels (**f**) (0.38 vs 0.73, p < 0.0001). Both metabolites showed significant correlation

Culture media supernatants were collected at 4 different time points throughout CD22 CAR T-cell manufacturing: Days 2, 3, 4, and 9. Our primary focus was on the findings on Day 2 and Day 9. Prior to transduction on Day 2, the pH of cell culture media ranged from 7.1–7.5 (Fig. 2a). pH level showed a statistically significant negative correlation with the D2 TNC count and fold expansion. On retrospective evaluation, we separated the products into 2 groups: 12 samples had relatively low pH on Day 2 (ranging from 7.093–7.289; mean 7.2), and 8 samples had high pH (ranging from 7.323–7.541; mean 7.4). The differences in pH levels between these 2 groups were statistically significant (p < 0.0001, Fig. 2a). The cell counts, fold expansion, and metabolic activity of T-cells were compared between the low pH and high pH groups. Cell products in the low pH group had significantly elevated total nucleated cell counts (414 vs 236 × 10^6^, p < 0.0061, Fig. 2b), greater fold expansion (0.85 vs 0.53, p < 0.0015, Fig. 2c), higher pCO_2_ (23.2 vs 17.6 mmHg, p < 0.001, Fig. 2d) and higher lactate levels (0.73 vs 0.38 g/L, p < 0.0001, Fig. 2f). Those in the low pH group also had reduced glucose levels (1.9 vs 2.3 g/L, p < 0.0003, Fig. 2e) compared with the high pH group. These results demonstrate the relationship between lower extracellular pH and T-cell growth, expansion, and glycolysis as pH levels correlated with pCO_2_, glucose, and lactate levels. On the other hand, Day 2 pH levels did not correlate with CD3% (Additional file 1: Supplementary Figure 2a) or pO_2_ levels (Additional file 1: Supplementary Figure 2b).

**Fig. 3:**
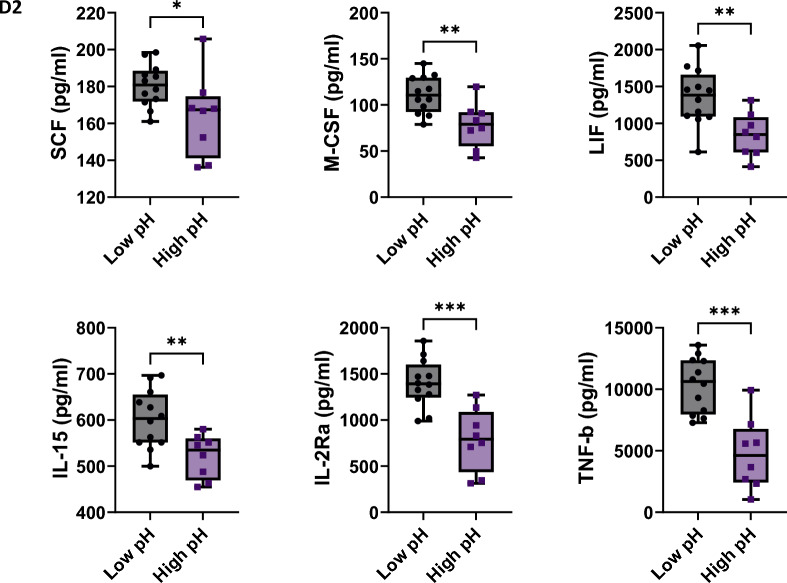
Elevated levels of selected cytokines and growth factors on Day 2 of the manufacturing process were detected in cell culture supernatants collected at low pH. Lower levels of extracellular pH induced increased secretion of SCF (p < 0.04), M-CSF (p < 0.004), LIF(p < 0.0052), IL-15 (p < 0.0066), IL2Ra (p < 0.0002) and TNF-b (p < 0.0001) into cell culture supernatants compared to the ones cultured in higher pH

Cytokine and growth factor levels were also compared between low and high pH groups. On Day 2 of the manufacturing process, six analytes demonstrated significantly greater levels among samples in the low pH group compared with the high pH group (Fig. 3). Increased levels of stem cell factor (SCF, p < 0.04), macrophage colony-stimulation factor (M-CSF, p < 0.004), leukemia inhibitory factor (LIF, p < 0.0052), interleukin 15 (IL-15, p < 0.0066), interleukin receptor 2 alpha (IL2Rα, p < 0.0002), and tumor necrosis factor beta (TNF-β, p < 0.0001) were observed in products with more acidic pH. In the context of increased proliferation associated with lower pH as shown above, these findings were consistent with our expectation that more active T-cells will secrete more cytokines and growth factors into the cell culture media.

### The pH of culture media at the end of manufacturing reflects metabolic profile, but does not correlate with expansion of final CAR T-cell products

**Fig. 4:**
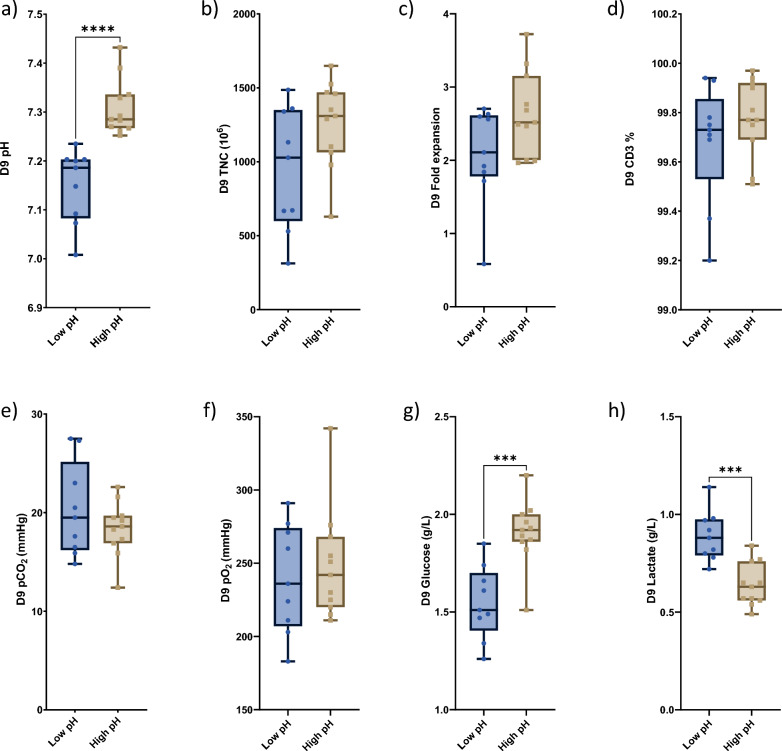
Increased pH levels at the harvest did not affect final TNC, fold expansion or gas tension. **a** Significant changes (p < 0.0001) in extracellular pH were noticed in CD22 CAR T-cell products at the harvest. 9 products were collected with pH values at about 7.1 and 11 with pH 7.3. Different pH values at the harvest did not affect final TNC levels (**b**), fold expansion (**c**), CD3 percentage (**d**), pCO_2_ (**e**), or pO_2_ (**f**). High pH was correlated with significantly upregulated glucose levels (p < 0.0003) (**g**) and negatively with lactate levels (p < 0.0002) (**h**)

Next, we investigated whether the pH level measured at the end of manufacturing was associated with cell or metabolic parameters. Overall, we observed a slight decline in pH values during the manufacturing process with higher values on Day 2 compared to Day 9. The Day 9 pH ranged from 7.0 to 7.4 and based on its values, the final CAR T-cell products were also separated into 2 groups representing low (9 patients, pH ranged from 7.008–7.235 with the mean pH of 7.1) and high pH (11 patients, pH ranged from 7.252 to 7.432 with mean pH of 7.3). The difference in final pH levels between these two groups was statistically significant (p < 0.0001, Fig. 4a). However, unlike the Day 2 pH groups, these final pH groups did not differ in terms of cell counts (Fig. 4b), fold expansion (Fig. 4c), CD3% (Fig. 4d), pCO_2_ (Fig. 4e), or pO_2_ (Fig. 4f). Higher pH at harvest correlated with higher glucose (1.91 vs 1.55 g/L; p < 0.0003, Fig. 4g) and lower lactate production (0.64 vs 0.89 g/L; p < 0.0002, Fig. 4h), suggesting reduced glycolysis and lower metabolic activity.

**Fig. 5:**
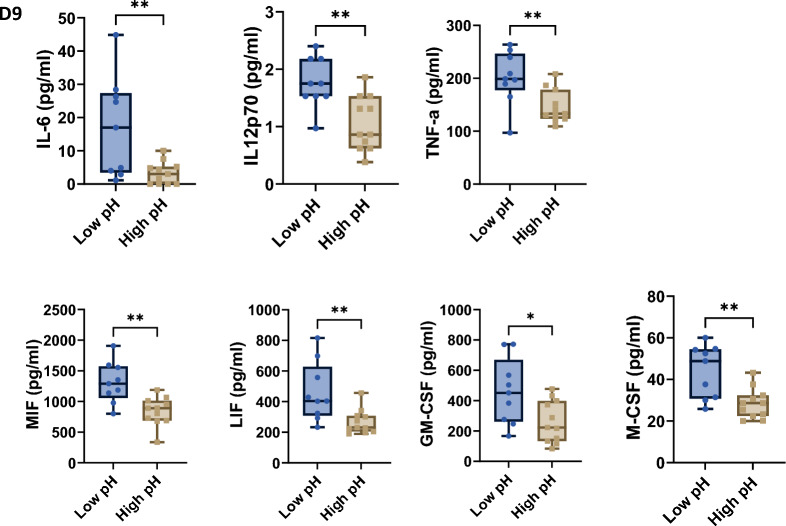
pH related changes in cytokines and growth factors in cell culture supernatants from CD22 CAR T-cells at the harvest. Reduced levels of IL6 (p < 0.008), IL-12p70 (p < 0.003), TNF-a (p < 0.008), LIF (p < 0.007), MIF (p < 0.002), M-CSF (p < 0.003) and GM-CSF (p < 0.02) were found in cell supernatants with higher pH

The Day 9 levels of 7 cytokines differed between the high and low pH groups. All 7 were elevated in the low pH group compared to the high pH group [interleukin 6 (IL6, p < 0.008), interleukin 12 subunit p70 (IL-12p70, p < 0.003), tumor necrosis factor alpha (TNF-α, p < 0.008), leukemia inhibitory factor (LIF, p < 0.007), macrophage migration inhibitory factor (MIF, p < 0.002), macrophage colony-stimulation factor (M-CSF, p < 0.003) and granulocyte macrophage colony-stimulation factor (GM-CSF, p < 0.02)] (Fig. 5). The levels of LIF and M-CSF were also greater in the low pH group on day 2 (Fig. 3), although others did not differ significantly.

### Sequencing profile of final CD22 CAR T-cells products demonstrates differences in T-cell activation pathways associated with pH

**Fig. 6:**
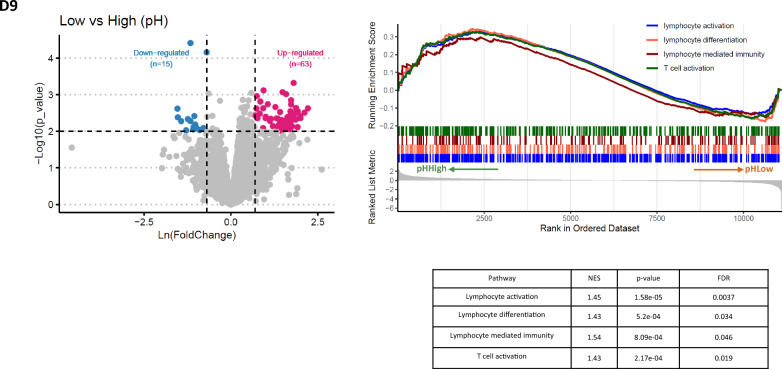
Gene expression changes in CD22 CAR T-cell products separated based on final pH. Gene expression analysis of the final products cultured either in high or low pH demonstrated in total 78 differentially expressed genes: 15 downregulated and 63 upregulated in low pH condition. Blue point represents downregulated genes. Red point indicates up-regulated genes. Genes with p value < 0.01 & |Foldchange|> 2 were considered as differentially expressed genes. GSEA analysis indicates the activation of the four pathways (lymphocyte activation and differentiation, lymphocyte mediated immunity and T cells activation) in high pH group. A significant positive NES value (with FDR < 0.05) is considered indicative of the activation state of the pathway in high pH group

CD22 CAR T-cell final product characteristics were analyzed by RNA sequencing (Fig. 6). Sequencing analysis of 17 final products assigned 78 differentially expressed genes between pH groups at harvest: 15 downregulated and 63 upregulated under low pH. GSEA analysis revealed that genes involved in T-cell activation, lymphocyte activation, differentiation, and mediated immunity, tend to be downregulated in low pH conditions. This highlights the value of pH as an indicator during CD22 manufacturing as it can help identify more activated CAR T-cell products (Table 2 shows the list of genes involved in each pathway).

**Table 2:** List of genes involved in each pathway

Lymphocyte activation	Lymphocyte differentiation	Lymphocyte mediated immunity	T cell activation
FCER1G	FCER1G	C4BPB	FCER1G
IGFBP2	KIT	FCER1G	IGFBP2
KIT	TCF7	RSAD2	KIT
TCF7	POU2AF1	MASP2	TCF7
POU2AF1	RSAD2	XCL1	RSAD2
RSAD2	CD79A	KLRC1	RELB
CD79A	RELB	CD8A	AIF1
RELB	LEF1	MALT1	LEF1
AIF1	CCR7	ZBTB1	CCR7
CXCR5	NTRK1	CD27	XCL1
CD38	CD83	CRTAM	TMIGD2
LEF1	GLI2	PRKCZ	CD83
CCR7	KLRC1	TNFSF4	SIRPG
XCL1	WNT4	SUSD4	GLI2
NTRK1	PRDM1	IL18RAP	TREML2
IRS2	CD8A	IL4R	CD8B
TMIGD2	MYB	LTA	KLRC1
CD83	FOXP1	THOC1	HHLA2
SIRPG	IRF4	IL23A	WNT4
GLI2	MALT1	IRF7	PIK3CA
TREML2	ZBTB1	ULBP2	PRDM1
CD8B	IL15	BATF	SLC11A1
KLRC1	CD27	TRAF6	CD8A
CD180	CRTAM	FOXP3	MYB
HHLA2	SOCS5	CD55	FOXP1
WNT4	HDAC5	ICAM1	IRF4
PIK3CA	RHOH	KLRC2	MALT1
TNFAIP3	DUSP10	NBN	ZBTB1
PRDM1	PRKCZ	PIK3R6	EBI3
SLC11A1	IL2RA	C1S	IL15
CD8A	EP300	IL20RB	CD27
MYB	CHD7	SLC22A13	WDFY4
FOXP1	IRF1	LGALS9	CTPS1
IRF4	TNFSF4	RFTN1	CRTAM
MALT1	RABL3	CEBPG	PELI1
ZBTB1	RIPK2	SMAD7	SOCS5
EBI3	TGFBR2	BCL3	RHOH
IL15	IL4R	NCR3	DUSP10
CD27	EZH2	SLC15A4	PRKCZ
WDFY4	GPR89A	AP1G1	IL2RA
CTPS1	MDK	CD160	CHD7
CRTAM	IL23A		IRF1
PELI1	KAT7		TNFSF4
SOCS5	KLF6		PAWR
HDAC5	CTLA4		IL6ST
RHOH	BATF		RABL3
DUSP10	SP3		RIPK2
PRKCZ	ITPKB		CD7
IL2RA	PPP2R3C		TGFBR2
LST1	RUNX1		IL4R
SLC39A10	VCAM1		GPR89A
EP300	ATG5		MDK
CHD7	FOXP3		IL23A
IRF1	PATZ1		CTLA4
TNFSF4	HDAC4		BATF
VAV3	DOCK10		NCK2
PAWR	PIK3R6		SP3
IL6ST	KAT2A		ITPKB
RABL3	TPD52		RUNX1
RIPK2	RUNX3		VCAM1
CD7	TP53		TRAF6
TGFBR2	IL6R		ATG5
IL4R	STAT3		AZI2
EZH2	ZMIZ1		FOXP3
GPR89A	LGALS9		PATZ1
MDK	PIK3CD		CD55
THOC1	PTPN2		MAPK8IP1
IL23A	NFKBIZ		ICAM1
KAT7	CEBPG		SLC7A1
KLF6	IRF2BP2		CBLB
ULBP2	NFATC2		CASP8
CTLA4	SMAD7		PIK3R6
BATF	IKZF3		KAT2A
NCK2	NOTCH2		IL20RB
SP3	ZC3H8		RUNX3
ITPKB	ZFP36L2		CD44
PPP2R3C			TP53
RUNX1			IL6R
VCAM1			STAT3
TRAF6			ZMIZ1
ATG5			LGALS9
AZI2			FYN
FOXP3			PIK3CD
PATZ1			PTPN2
ZNF335			DLG5
CD55			SRC
MAPK8IP1			NFKBIZ
ICAM1			DPP4
HDAC4			NFATC2
SLC7A1			SMAD7
CHRNB2			SDC4
KLRC2			ZC3H8
DOCK10			ZFP36L2
CBLB			PAG1
NBN			PAK1
CASP8			SEMA4A
PIK3R6			BCL3
KAT2A			
TPD52			
IL20RB			
RUNX3			
CD44			
TP53			
IL6R			
STAT3			
ZMIZ1			
TNIP2			
LGALS9			
FYN			
PIK3CD			
PTPN2			
DLG5			
SRC			
NFKBIZ			
DPP4			
CEBPG			
IRF2BP2			
NFATC2			
SMAD7			
IKZF3			
NOTCH2			
SDC4			
AHR			
ZC3H8			
ZFP36L2			
PAG1			
BANK1			
PAK1			
SEMA4A			
BCL3			
NCR3			
ST3GAL1			
IL21R			
SLC15A4			
AP1G1			
JAK3			

### Low pH at D2 is associated with clinical outcome

**Fig. 7:**
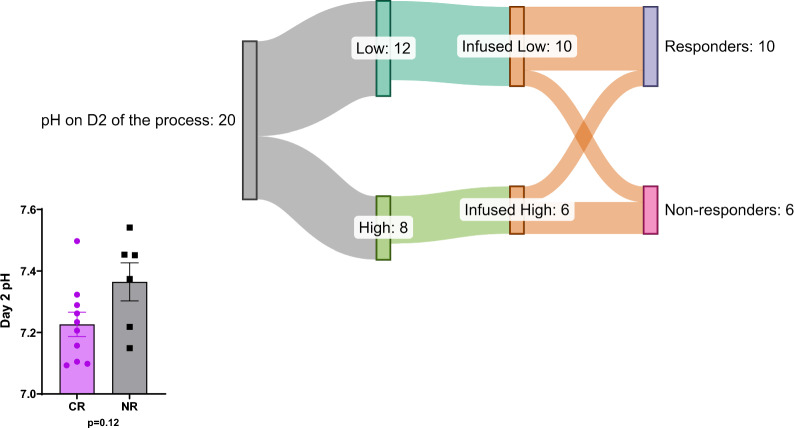
D2 pH and its effect on clinical outcome of CD22 CAR-T cells. 10 CD22 CAR-T cells that start manufacturing process with low pH, resulted in 8 responders and 2 non-responders. On the other hand, 6 products that manufacturing process started with high pH, revealed only 2 responders and 4 non-responders

To determine if product pH on Day 2 correlated with clinical outcomes, we compared disease response rates between the low and high pH groups (Fig. 7). Of the 12 patients whose samples were in the low pH group on D2, 10 were infused and 2 were not due to progressive disease. Among the patients receiving the cells, 8 (80%) achieved a complete response (CR), which is defined as complete bone marrow response at 28 days. The high pH group contained 8 patients. Six patients received CAR T-cell infusions and 2 did not due to rapidly progressive disease. In contrast to the low pH group, among those with high pH, only 2 patients (33%) achieved a clinical response, while 4 (67%) did not. The relationship between low starting pH and CR was most consistent in the subsets of patients with high baseline disease burden (M3 marrow, > 25% blasts) (Additional file 1: Supplemetary Figure 3a) and prior hematopoietic stem cell transplant (HSCT) (Additional file 1: Supplemetary Figure 3b).

Moreover, we observed a difference in toxicity patterns between the low and high pH groups. In the low pH group, 8 patients (all those with CR) developed cytokine release syndrome (CRS), 5 (62.5%) of them with Grade 1 and 3 with (37.5%) Grade 2. Non-responders in this group did not have any toxicities. Only 25% of the patients with clinical response in the low pH group experienced neurotoxicity and 50% had IEC-HS (immune-effector cell HLH-like syndrome) (Table 3). In the high pH group, both patients with clinical response developed Grade 2 CRS, neurotoxicity, and IEC-HS. Furthermore, in the high pH group, all non-responders experienced toxicities as well: all had CRS, 2 of them had neurotoxicity, and one had IEC-HS.

**Table 3:** pH correlation to clinical outcome and adverse events

Patient	pH	Clinical response	CRS grade^a^	IEC-HS	Neurotoxicity
5	Low	–	–	–	–
18	Low	–	–	–	–
11	Low	No	0	No	No
13	Low	No	0	No	No
3	Low	**Yes**	2	Yes	No
4	Low	**Yes**	1	No	No
7	Low	**Yes**	1	Yes	No
8	Low	**Yes**	1	No	No
12	Low	**Yes**	1	Yes	No
16	Low	**Yes**	2	No	Yes
19	Low	**Yes**	2	Yes	No
20	Low	**Yes**	1	No	Yes
10	High	–	–	–	–
17	High	–	–	–	–
6	High	No	1	No	No
9	High	No	2	Yes	Yes
14	High	No	1	No	Yes
15	High	No	1	No	No
1	High	**Yes**	2	Yes	Yes
2	High	**Yes**	2	Yes	Yes

*CRS* Cytokine Release Syndrome, *IEC-HS* Immune-Effector Cell HLH-like syndrome

^a^CRS graded based on American Society for Transplantation and Cellular Therapy criteria: Lee [28]

**Fig. 8:**
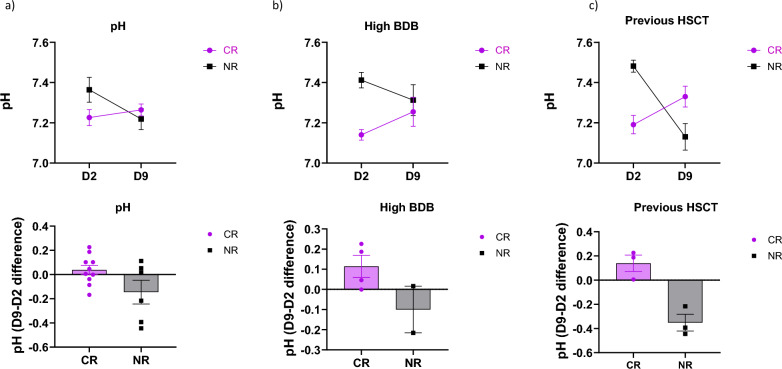
pH connection to clinical outcome. **a** Association between low pH at the beginning of the manufacturing process and clinical response has been proven in 80% of the patients whereas only 20% of patients demonstrated clinical response when their cells were manufactured in higher pH. **b** Patients with high baseline disease burden which cells were kept at lower pH displayed higher probability of clinical response. **c** Patients who received previous hematopoietic stem cell transplant were associated with clinical responsiveness as long as the manufacturing process of their cells started with low pH

Analysis of pH changes over the course of manufacturing demonstrated that patients who achieved CR were more likely to have cells with stable or slightly increased pH values during the manufacturing process, whereas those who did not have CR had a drop in pH (Fig. 8a). Similar to the pattern observed with starting pH, the association between a drop in pH and lack of clinical response was most pronounced among patients with high baseline disease burden or history of prior HSCT (Fig. 8b and c).

## Discussion

The aim of this study was to assess the relationship between culture conditions, specifically culture pH, and the growth kinetics and properties of CD22 CAR T-cells used for the treatment of pediatric B-cell acute lymphoblastic leukemia. Our data demonstrate that pH fluctuations during the CAR T-cell manufacturing process correlate with properties of the cellular product and may be indicative of clinical outcome. Based on our retrospective analysis, high pH at the start of manufacturing is associated with reduced number of viable nucleated cells, slowed cell expansion, and reprogrammed T-cell metabolism. Patients whose cells had a high pH at the start of the manufacturing tended to have a lower probability of clinical response. Since the data suggests that pH levels at 7.2 to 7.3 are indicative of optimal manufacturing conditions, the measure may be useful as an predictor of appropriate T-cell growth, differentiation, and clinical efficacy. We therefore propose that careful monitoring of pH during the manufacturing of cellular products is critical, and accurate pH monitoring during cGMP manufacturing should become a standard.

In our samples, lower pH generally correlated with a picture of increased T-cell proliferation. Lower pH was connected with a drop in glucose and an increase in lactate levels [20, 21]. This shows that T-cells grown in lower pH are more metabolically active compared to high pH. As the most rapid cell expansion occurs during the early days of CAR T-cell manufacturing, the Day 2 pH remains a better indicator of this process than the Day 9 pH, which is reflecting the media typically after several days of slower growth. It will be important in future studies to explore whether cell expansion patterns in vitro are recapitulated in vivo after cells are infused.

Lower pH may not only be a consequence of greater T-cell expansion but may actually be supporting further growth. Despite T-cell dysfunction in truly acidic environments, pH in the lower range of normal has been reported to be beneficial in the context of CAR T-cell expansion. In in vitro studies, reduction in extracellular pH to 6.6 significantly affected not only T cell proliferation but also reduced IL-2 and IFN-γ secretion, downregulated CD25 and CD71 expression, and upregulated CTLA4 expression leading to apoptosis [15]. Similarly, pH in the range of 6.0 to 6.5 has been shown to induce anergy in CD8+ T lymphocytes, with impaired proliferation, reduced cytokine production, decreased CD25 expression, and inhibition of STAT5 and p-ERK activation [16]. Although the pH fluctuations in our study might appear minor, the results are consistent with other studies showing that only small variations in culture pH can have a substantial effect on T-cells. Others have reported improved T-cell stimulation, expansion, and proliferation in lower pH, 7.0–7.3, compared to neutral pH [22, 23], which may be consistent with our findings. Similarly, only small variations in pH were also shown to be critical in hematopoietic cultures, mainly for progenitor cell differentiation and cloning efficiency [24].

In addition to predicting T-cell proliferation, the pH of culture medium may also help predict cytokine production. We demonstrated that cells starting out in lower pH had increased production of several cytokines including IL-6, IL12p70, TNF-a and GM-CSF, which play an important role not only in CAR T-cells’ targeted cytotoxicity, but also in development of off-target effects, such as CRS and neurotoxicity [25–27]. Further analysis of cytokine production during CAR T-cell manufacturing may yield important insight into which patients are at risk for developing CAR T-cell associated toxicities.

There are important limitations to this exploratory study, and further investigation will be needed to confirm the trends reported here. The sample size is limited, and since our study included only anti-CD22 CAR T-cells, the findings might not be applicable to other CAR T-cell types. Overall, the relationship between pH and T-cell characteristics likely varies with the specific manufacturing protocol, including culture media, manufacturing platform, and timing. We are, however, continuing to collect more samples as new patients are treated, including those on the ongoing CD22 trial and trials of other novel CAR T-cell products. Importantly, the data reported here were collected over the course of our standard CAR T-cell manufacturing process, and no intentional adjustments to pH were made. Therefore, we can report only correlations with other parameters rather than any causal relationships. Future work will focus on pH modification during the manufacturing process to determine whether this will have any effect on the characteristics and efficacy of the final product.

Currently there are no guidelines or standards advising monitoring of pH during cell culture for CAR T-cell manufacturing. The emerging data, however, points to pH as an important and easily accessible tool for monitoring and standardizing CAR T-cell production. Furthermore, it has potential to serve as an early indicator of clinical outcome. Since adjusting the pH of cell culture media can be a relatively straightforward and inexpensive intervention, further prospective studies are needed to determine whether actively controlling pH during manufacturing is beneficial.

## Conclusions

Measurements of pH throughout CAR T-cell manufacturing correlate with cell proliferation as well as other metabolic properties of CAR T-cells. Accurate pH tracking during CAR T-cell manufacturing may serve as an important metric of the quality of the cellular product and may become a predictive marker of CAR T-cell clinical efficacy. pH should be monitored throughout CAR T-cell manufacturing, and combined with other factors, it could significantly improve the quality, compatibility, and safety of CAR T-cells.

### Supplementary Information

**Additional file 1: Figure S1.** Manufacturing schema for CD22 CAR T-cells and simplified representative gating strategy of the final product. a) Simplified culture schematic with major processing steps during CD22 CAR T-cells manufacturing at Center for Cellular Engineering, Clinical Center, NIH. b) CD56, CD14, and CD15% in the apheresis bag and selected CD4/CD8 T cells. c) Representative flow cytometry staining including protein L gating strategy in final CD22 CAR T product. Viable cells (7-AAD negative population) were initially gated from the singlets and CD45^+^ cells. Viable CD3^+^ cells were then gated and analyzed for protein L expression. The untransduced cells were stained at the same time and were used to identify protein L^+^ population in transduced cells. **Figure S2.** CD3% and pO_2_ levels did not correlate with D2 pH values. a) pH did not impact % of CD3 (87% for low pH vs 89% for high pH) and showed no correlation with pH on D2 of the CD22 CAR T-cells manufacturing process. b) No obvious changes were observed in oxygen levels comparing cultures with low or high pH. pO_2_ did not correlate with pH on D2 of the CD22 CAR T-cells manufacturing process. **Figure S3.** The relationship between low pH and CR in patients with high baseline disease burden and prior HSCT. a) Of the 6 patients with M3 baseline disease burden, the 4 who started out with low pH achieved a complete response, whereas the two patients with high disease burden who started out with higher pH had no response. b) A similar pattern was noted in patients that have previously received a hematopoietic stem cell transplant, but not among those who had not been exposed to transplant.

## Data Availability

Patient-related data not included in the paper might be subject to patient confidentiality. Raw sequencing data in this study are available with reasonable request to the corresponding authors.

## Abbreviations

GMP: Good Manufacturing Practice
CAR: Chimeric antigen receptor
ALL: Acute lymphoblastic leukemia
FDA: Food and Drug Administration
CCE: Center for Cellular Engineering
TNC: Total nucleated cell
GSEA: Gene Set Enrichment Analysis
HSC: Hematopoietic stem cell
HSCT: Hematopoietic stem cell transplant
CRS: Cytokine release syndrome
CR: Complete response
L: Lentivirus
TR: Transduction
CHM: Change of media
