# Supplementary material for: Deciphering the importance of culture pH on CD22 CAR T-cells characteristics
Source: J Transl Med. 2024 Apr 24;22:384. doi: 10.1186/s12967-024-05197-5 (PMC11043048; doi:10.1186/s12967-024-05197-5)
Supplement: Supplementary file 1 — Additional file 1: Figure S1. Manufacturing schema for CD22 CAR T-cells and simplified representative gating strategy of the final product. a) Simplified culture schematic with major processing steps during CD22 CAR T-cells manufacturing at Center for Cellular Engineering, Clinical Center, NIH. b) CD56, CD14, and CD15% in the apheresis bag and selected CD4/CD8 T cells. c) Representative flow cytometry staining including protein L gating strategy in final CD22 CAR T product. Viable cells (7-AAD negative population) were initially gated from the singlets and CD45+ cells. Viable CD3+ cells were then gated and analyzed for protein L expression. The untransduced cells were stained at the same time and were used to identify protein L+ population in transduced cells. Figure S2. CD3% and pO2 levels did not correlate with D2 pH values. a) pH did not impact % of CD3 (87% for low pH vs 89% for high pH) and showed no correlation with pH on D2 of the CD22 CAR T-cells manufacturing process. b) No obvious changes were observed in oxygen levels comparing cultures with low or high pH. pO2 did not correlate with pH on D2 of the CD22 CAR T-cells manufacturing process. Figure S3. The relationship between low pH and CR in patients with high baseline disease burden and prior HSCT. a) Of the 6 patients with M3 baseline disease burden, the 4 who started out with low pH achieved a complete response, whereas the two patients with high disease burden who started out with higher pH had no response. b) A similar pattern was noted in patients that have previously received a hematopoietic stem cell transplant, but not among those who had not been exposed to transplant. [file 12967_2024_5197_MOESM1_ESM.pptx]

## Slide 1
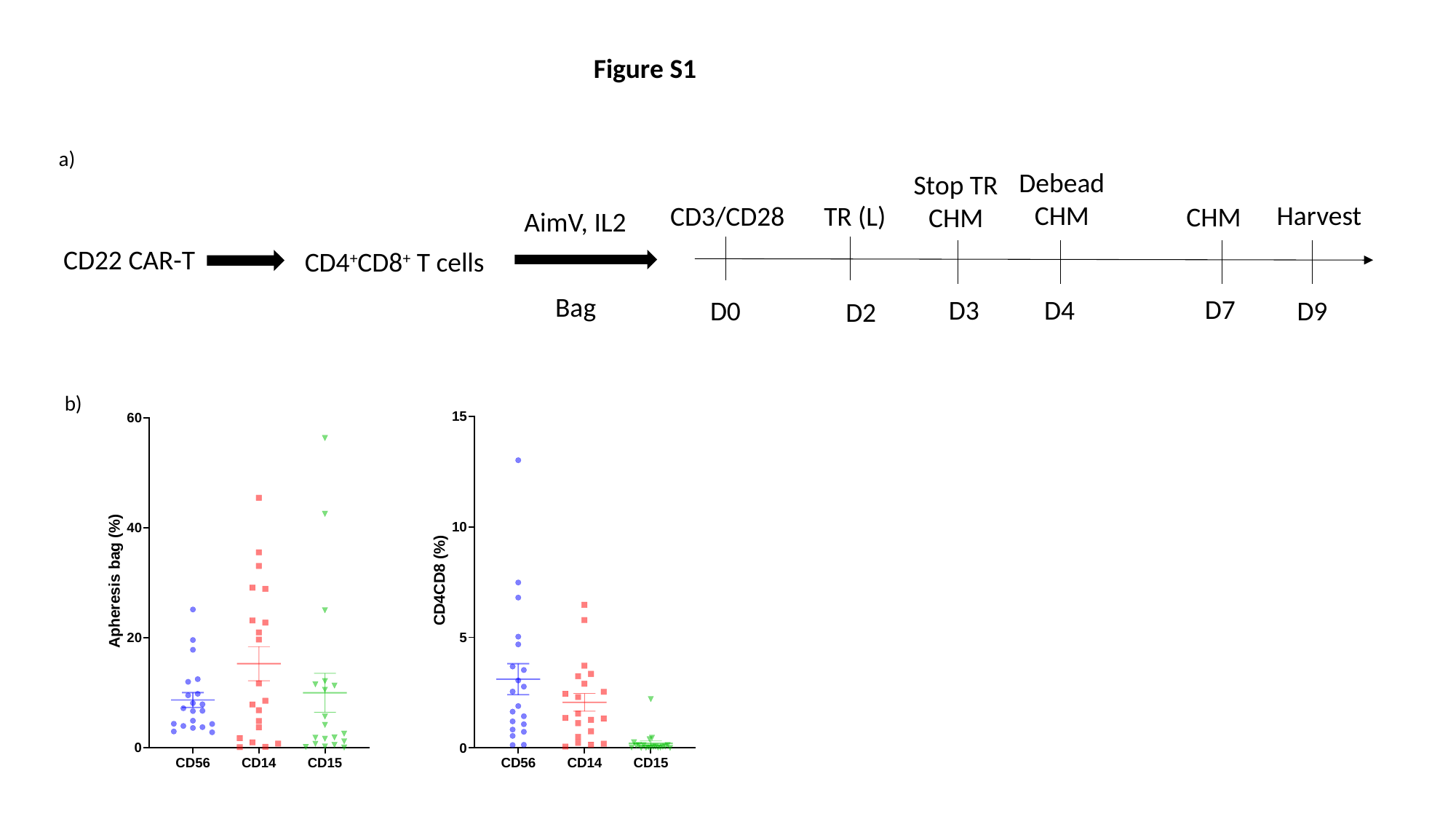

Figure S1
a)
Debead
CHM
Stop TR
CHM
Harvest
TR (L)
CD3/CD28
CHM
AimV, IL2
CD22 CAR-T
CD4+CD8+ T cells
Bag
D7
D4
D3
D9
D0
D2
b)

## Slide 2
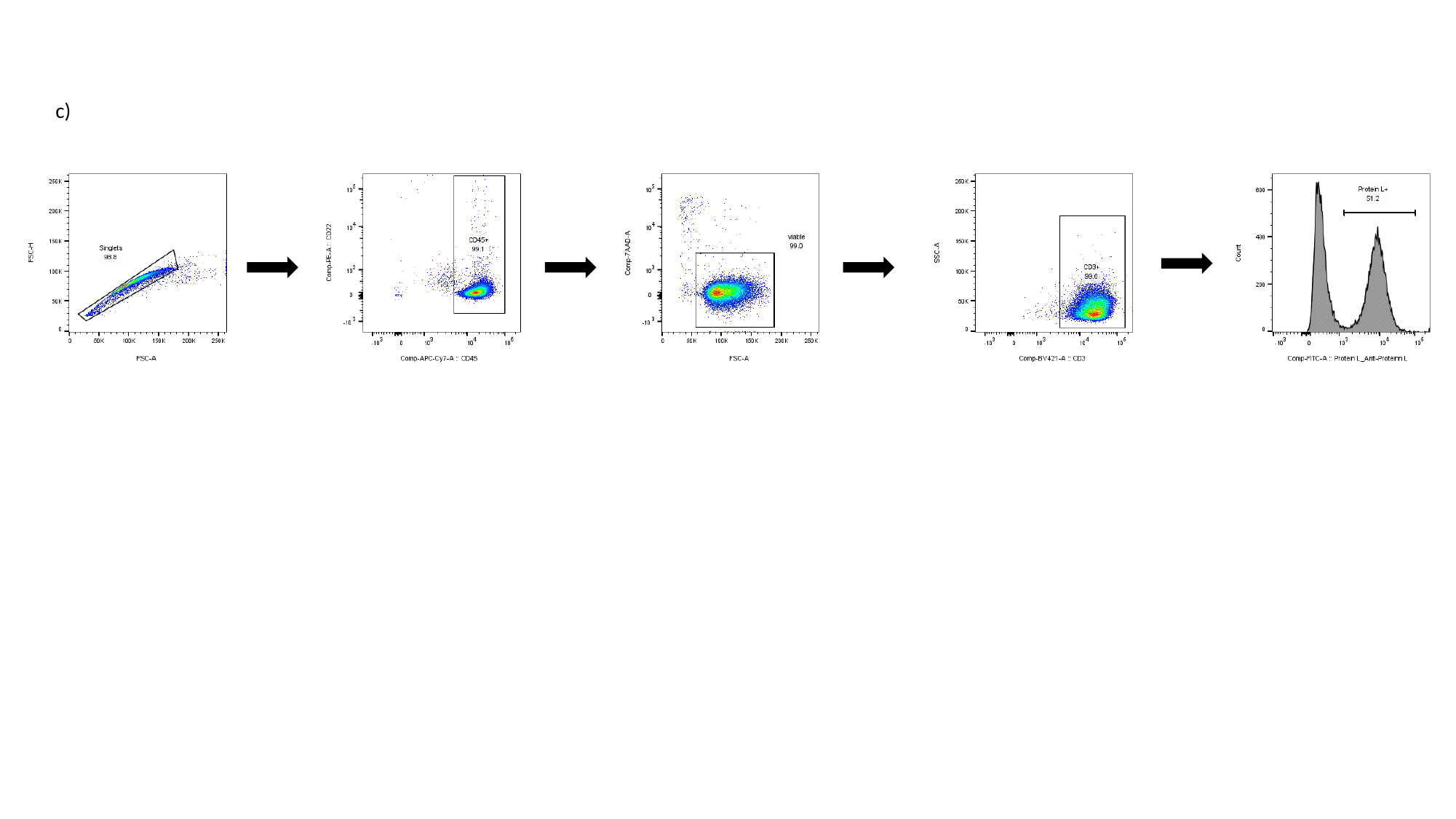

c)

## Slide 3
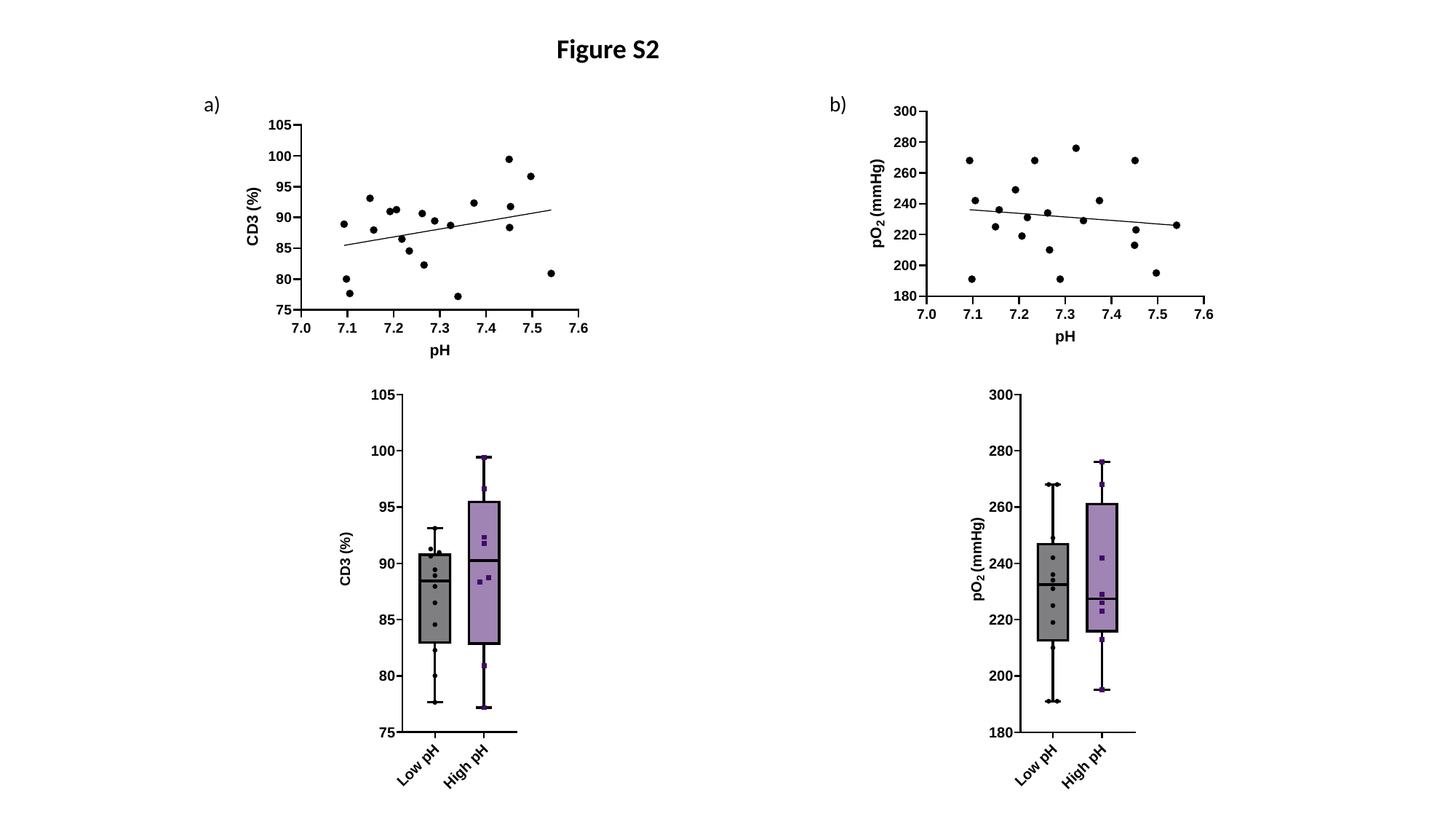

Figure S2
a)
b)

## Slide 4
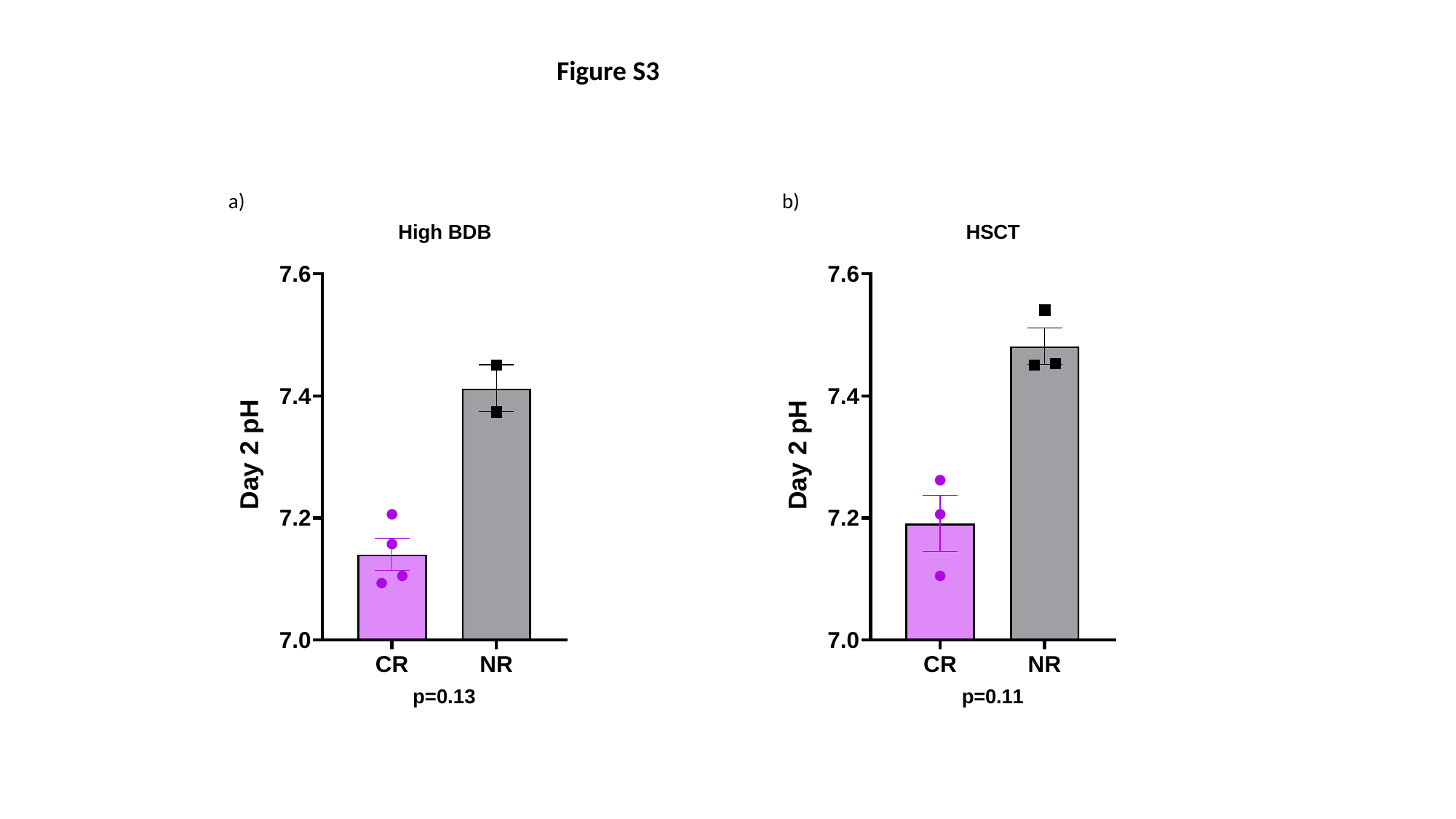

Figure S3
a)
b)
